# HMG20A was identified as a key enhancer driver associated with DNA damage repair in oral squamous cell carcinomas

**DOI:** 10.1186/s12903-022-02500-y

**Published:** 2022-11-05

**Authors:** Li Na, Zhang Meijie, Zhai Wenjing, Zhou Bing, Duan Yanhao, Liu Shanshan, Qiu Yongle

**Affiliations:** 1 Department of Stomatology, Second Hospital of Shijiazhuang, 050000 Shijiazhuang, Hebei China; 2 Department of Stomatology, Qinhuangdao Hospital of Traditional Chinese Medicine, 066000 Qinhuangdao, Hebei China; 3 Department of Stomatology, People’s Hospital of Shijiazhuang, 050000 Shijiazhuang, Hebei China; 4 Department of Stomatology, Cangzhou People’s Hospital, 061001 Cangzhou, Hebei China; 5 Department of Stomatology, Fourth Affiliated Hospital, Hebei Medical University, 12 Health Road, 050017 Shijiazhuang, Hebei China

**Keywords:** Oral squamous cell carcinoma, DNA repair, Enhancer driver, Metastasis, High mobility group 20A

## Abstract

**Background:**

Oral squamous cell carcinoma (OSCC) is the main type of oral cancer. Disturbing DNA repair is an invaluable way to improve the effectiveness of tumor treatment. Here, we aimed to explore the key enhancer drivers associated with DNA damage repair in OSCC cells.

**Methods:**

Gene Set Enrichment Analysis (GSEA), Gene Set Variation Analysis (GSVA) and Kaplan-Meier analysis were applied to explore the relationship among DNA repair-related genes expression and clinical phenotypes based on The Cancer Genome Atlas (TCGA) database. HOMER software and Integrative Genomics Viewer were applied to identify and visualize enhancers using GSE120634. Toolkit for Cistrome Data Browser was applied to predict transcription factors. Human Protein Atlas Database was used to analyze the protein levels of transcription factors in OSCC and control tissues. Seventy-two OSCC patients were included in this study. qRT-PCR was used to detect transcription factor expression in OSCC and adjacent control tissues collected in this study. qRT-PCR and ChIP-qPCR were used to verify the binding of transcription factors to enhancers, and regulation of target genes transcription. Transcription factor knockdown and control cells were treated with cisplatin. CCK8 was used to detect cell viability and proliferation. Western blotting was implemented to detect the levels of DNA repair-related proteins. Transwell assay was used to detect cell invasion.

**Results:**

DNA repair was positively associated with the OSCC metastatic phenotype. Patients in the cluster with high expression of DNA repair-related genes had a worse prognosis and a higher proportion of advanced stage, low-differentiation, alcohol consumption and smoking compared to the cluster with low DNA repair-related gene expression. Seventeen metastasis-specific enhancer-controlled upregulated DNA repair-related genes, with the top two upregulated genes being ADRM1 26 S proteasome ubiquitin receptor (ADRM1) and solute carrier family 12 member 7 (SLC12A7) were screened. High mobility group 20 A (HMG20A) was the key prognostic enhancer driver regulating metastasis-specific DNA repair-related genes, with higher expression in OSCC tissues than normal control tissues, and higher expression in metastatic OSCC tissues than non-metastatic OSCC tissues. HMG20A bound to the metastasis-specific enhancers of ADRM1 and SLC12A7, thereby promoting ADRM1 and SLC12A7 expression. Knockdown of HMG20A enhanced cisplatin sensitivity of cells, and inhibited OSCC cells from repairing DNA damage caused by cisplatin, as well as proliferation and invasion of OSCC cells.

**Conclusion:**

HMG20A was identified as the key prognostic enhancer driver regulating DNA repair in OSCC cells, providing a new therapeutic target for OSCC.

**Supplementary Information:**

The online version contains supplementary material available at 10.1186/s12903-022-02500-y.

## Introduction

Oral squamous cell carcinoma (OSCC) is a highly prevalent head and neck malignancy worldwide [1, 2]. Tobacco smoking, alcohol consumption, HPV infection and genetic factors, etc. are risk factors for OSCC [1, 3]. The five-year survival for patients with Tumor-Node-Metastasis (TNM) stage I/II OSCC is ~ 90%, whereas the five-year survival for patients with TNM stage III/IV OSCC is only ~ 30% [4, 5. Most OSCC patients (> 66%) are diagnosed at TNM stage III/IV [6]. Cervical lymph nodes and distant metastases are the major causes of poor prognosis in advanced OSCC [3, 7]. Patients with OSCC are often treated with a multidisciplinary approach, including surgery, chemotherapy, biotherapy and radiotherapy [6]. Despite recent advances in the diagnosis and treatment of OSCC, the overall survival of OSCC still has not improved significantly. Therefore, the development of effective treatment options for OSCC is urgently needed.

DNA damage leads to apoptosis, autophagy, senescence and other responses [8, 9]. DNA repair pathways include five main types, including base excision repair, nucleotide excision repair, mismatch repair, homologous recombination and non-homologous end-joining [10]. Abnormal DNA damage repair capacity of tumor cells contributes the maintenance of malignant phenotype, and the acquisition of resistance to radiotherapy and chemotherapy [11]. Therefore, blocking DNA repair pathway is an important approach for tumor treatment. For instance, cisplatin, the first-line chemotherapeutic drug for OSCC, induces DNA damage through the formation of cisplatin-DNA adducts, leading to cell cycle arrest and apoptosis [12]. Nucleotide excision repair is a key pathway to resist damage caused by cisplatin [13]. ERCC excision repair 1, endonuclease non-catalytic subunit (ERCC1) is an important factor of nucleotide excision repair pathway [13]. It was found that high ERCC1 expression is associated with cisplatin resistance and poor prognosis in head and neck squamous carcinoma [14]. DNA double-strand breaks are a serious form of DNA damage [15]. γ-H2AX marking is observed at DNA double-strand break sites, and the level of γ-H2AX is positively correlated with the degree of DNA damage [16]. γ-H2AX recruits DNA repair-related proteins, particularly BRCA1 DNA repair associated (BRCA1) and RAD51 recombinase (RAD51), to activate the homologous recombination repair pathway [16]. Wang et al. found that palbociclib inhibits DNA damage repair in OSCC cells by suppressing RAD51 expression [17]. Despite the progress made in the study of DNA repair in OSCC, the mechanisms of DNA repair in OSCC cells and their relationship with the malignant phenotype remain to be clarified. Exploring the mechanisms of DNA repair in OSCC cells may provide guidance to improve the effectiveness of OSCC treatment.

Enhancers are typically 200–1500 bp in size which bind to transcription factors and cofactors to *cis*-regulate the expression of target genes [18]. Enhancers can be located at the 3’-end, 5’-end and intron of target genes. In addition, enhancers can remotely regulate the expression of target genes. Enhancer activation is characterized by high levels of acetylation of histone 3 at lysine 27 (H3K27ac) and histone 3 lysine 4 monomethylation (H3K4me1), and is cell-specific [18, 19]. In contrast, poised state enhancers repress target gene transcription and generally characterized by both H3K4me1 and histone 3 lysine 27 trimethylation (H3K27me3) histone tags [20–23]. Therefore, H3K27ac is defined as a specific enhancer activation signal. Enhancers are closely associated with the development of many cancers, including OSCC [24]. To our best knowledge, the mechanisms by which enhancers regulate genes related to DNA repair in OSCC are still incompletely understood.

The overall aim of the current study was to uncover the key enhancer drivers affecting DNA repair in OSCC cells. We analyzed the relationship between DNA repair-related genes and clinical features. Subsequently, DNA repair-related genes regulated by metastasis-specific enhancers as well as their key transcription factor with prognostic value were screened. Finally, the function of the key enhancer driver was explored in vitro. This study is expected to identify novel DNA repair blocking targets to support the advancement of OSCC clinical treatment efficiency.

## Methods

### Data collection

Gene expression and the corresponding clinical data of 28 metastatic OSCC (metastatic recurrence occurred within 5 years following surgery) and 47 non-metastatic OSCC (without metastasis within 5 years following surgery) patients were downloaded from The Cancer Genome Atlas database (TCGA, https://portal.gdc.cancer.gov/). The “edgeR” package in R software was employed to normalize the gene expression data. H3K27ac ChIP-seq data of GSE120634 cohort were downloaded from the Gene Expression Omnibus database (GEO, http://www.ncbi.nlm.nih.gov/geo/). Immunohistochemical data of OSCC and normal control tissues were downloaded from The Human Protein Atlas database (https://www.proteinatlas.org/).

### Gene Set Enrichment Analysis (GSEA) and Gene Set Variation Analysis (GSVA)

GSEA (http://www.broadinstitute.org/gsea/index.jsp) was performed to unearth the underlying relationship among DNA repair and metastatic phenotype of OSCC using data downloaded from TCGA. Six DNA repair-related gene sets (“DNA repair”, “base excision repair”, “nucleotide excision repair”, “mismatch repair”, “homologous recombination” and “non-homologous end-joining”) were obtained from the Molecular Signatures Database (MSigDB, https://www.gsea-msigdb.org/gsea/msigdb/index.jsp). Normalized (NOM) P < 0.05, normalized enrichment score (NES) ≥ 1 and false discovery rate (FDR) q-value ≤ 0.25 was considered as screening criteria for significant enrichment.

Hierarchical clustering was applied to cluster OSCC samples from TCGA based on the expression of DNA repair-related genes. Hierarchical clustering was shown with a dendrograms in heatmaps. DNA repair pathway enrichment scores for each cluster were calculated using “GSVA” package in R software. Comparisons of enrichment scores among clusters were performed using one-way analysis of variance (ANOVA) followed by Turky’s test.

### Analysis of differentially expressed genes

DNA repair-related genes in cluster 2 and cluster 3 were analyzed for differential expressed based on TCGA-OSCC data. Differential gene expression analysis was performed using the “limma” package in R software. Genes with |log2 fold change| ≥1.0 and Benjamini-Hochberg adjusted P < 0.05 were retained as the differentially expressed genes.

### Prognostic analysis of different clusters and transcription factors

Prognostic analysis was performed by Kaplan-Meier plots and log-rank tests using data downloaded from TCGA. Disease-free survival (DFS) analysis was performed in each cluster using the “survival” R package. Transcription factors were predicted using The Toolkit for Cistrome Data Browser (http://dbtoolkit.cistrome.org/). Transcripts per million (TPM) < 1 was used to reject transcription factors which were low expressed in OSCC. Hazard ratios (HRs) and 95% confidence intervals (CIs) were calculated using the “survival” R package. OSCC patients were divided into high and low expression groups based on the quartiles. Overall survival was evaluated using the “survival” R package.

### Enhancer identification

H3K27ac ChIP-seq data of HN120Pri and HN120Met cells from GSE120634 data set were analyzed for enhancer identification using “findPeaks” tool in HOMER software. Integrative Genomics Viewer (https://igv.org) was applied to visualize H3K27ac peaks. In this study, the gene closest to an enhancer locus on the genome was identified as an enhancer-controlled gene. The “annotatePeaks” tool in HOMER software was conducted to screen the enhancer-controlled genes.

### Cell lines and cell culture

BHY and HSC3 were two human metastatic OSCC cell lines, which were purchased from the Japanese Collection of Research Bioresources (JCRB). All cells were cultured in Dulbecco’s Modified Eagle’s Medium (DMEM, Gibco, USA) containing 10% fetal bovine serum (Gibco, USA) at 37 °C and 5% CO_2_.

### Cell transfection

SiRNA specifically targeting high mobility group 20 A (HMG20A) (si-HMG20A) and the control si-RNA (si-NC) were synthesized by Gene Pharma (Shanghai, China). BHY and HSC3 cells were seeded into 6-well plates at the density of 1 × 10^5^ cells per well, and transfected with si-HMG20A or si-NC using Lipofectamine 3000 (Invitrogen, USA) following the manufacturer’s instructions. The transfection concentration of si-RNAs was 50 nM/1 × 10^5^ cells. The sequence of si-RNAs were as follows: si-HMG20A, 5′-AGGCAAAUCUCAUAGGCAA-3′ [25; si-NC, 5′-GCACAAGCUGGAGUACAACUACATT-3′. The transfected cells were processed for subsequent studies 48 h after transfection.

### Patient collection

A total of 72 patients with pathologically confirmed OSCC at the Fourth Affiliated Hospital of Hebei Medical University were collected in this study (January 2015 to December 2016). These OSCC patients were further divided into non-metastatic OSCC patients (n = 40) and metastatic OSCC patients (n = 32) according to whether metastasis occurred within 5 years after surgery. All included patients had not undergone any cancer-related treatment prior to surgery. OSCC tissue and adjacent control tissue samples from all participants were preserved in the form of frozen specimens.

This study was approved by the Ethics Committee of the Fourth Affiliated Hospital of Hebei Medical University (Shijiazhuang, China). All participants were provided with written informed consent prior to the start of the study.

### qRT-PCR

Total RNA was extracted from cells and tissues using TRIzol reagent (Invitrogen, USA). 1 µg of total RNA was used to synthesize cDNA by High-Capacity cDNA Reverse Transcription Kit (Thermo Fisher, USA). qRT-PCR was conducted using the Power SYBRs Green PCR Master Mix (Thermo Fisher, USA), and executed by ABI 7900 real-time PCR system (Applied Biosystems, USA). The 2^−ΔΔCt^ method was applied to determine the relative expression levels with GAPDH as the endogenous control. Primers used in qRT-PCR as following: ADRM1 26S proteasome ubiquitin receptor (ADRM1)-F: 5’-GGCGGGAAAGATGTCCCTG-3’; ADRM1-R: 5’-GTCGTCCGTCTGCTGAATGT-3’. Solute carrier family 12 member 7 (SLC12A7)-F: 5’-CTGGCGGGTCCTACTACATGA-3’; SLC12A7-R: 5’-AAAATCTCGATGGTCCCCAAAAT-3’. HMG20A-F: 5’-ATGACTAGCTCCACCCTACCG-3’; HMG20A-R: 5’- CTCGTTTACTTCGTTGCTCATCT-3’. GAPDH-F: 5’-GGAGCGAGATCCCTCCAAAAT-3’; GAPDH-R: 5’-GGCTGTTGTCATACTTCTCATGG-3’.

### Chromatin immunoprecipitation (ChIP)-qPCR

HSC3 and BHY cells transfected with si-NC or si-HMG20A were cross-linked by 1% formaldehyde for 10 min at room temperature. The cross-linked cells were lysed with cell lysis solution for 20 min, and then sonicated using Covaris E220 (Woburn, USA) followed by immunoprecipitation with anti-H3K27ac (ab4729, Abcam, USA) at 4°C overnight. Gel Extraction Kit (Omega Bio-Tek, USA) was used to purify DNA. Finally, the purified products were subjected to qRT-PCR. The primers of enhancer regions of ADRM1 and SLC12A7 as following: ADRM1-E1-F: 5’- CCTCACAGCACAAGCTCAGA-3’; ADRM1-E1-R: 5’- ACCTTAATGGCTGCAGGACC-3’. SLC12A7-E1-F: 5’- CGGATGGAAGGGCCTAAGAG-3’; SLC12A7-E1-R: 5’- CCATCCTGGCTCCAAATCCC-3’.

### Western blotting

Total protein of BHY and HSC3 cells was extracted using RIPA buffer (Sigma-Aldrich, USA). Protein concentration was measured using the Pierce BCA protein assay (Thermo Fisher, USA). Proteins were subjected to SDS-PAGE for separation, followed by transfer to PVDF membranes (Millipore, USA). After immersion in 5% nonfat milk for 1 h, the membranes were cut according to the molecular weight of the protein and incubated with primary antibodies, anti-HMG20A (1:2000, 12085-2-AP, Proteintech, USA), anti-β-actin (1:2000, ab8226, Abcam, USA), anti-ERCC1 (1:2000, ab129267, Abcam, USA) and anti-γ-H2AX (1:2000, ab81299, Abcam, USA), overnight at 4 °C, followed by incubating with goat anti-rabbit IgG H&L (1:5000, ab96899, Abcam, USA) at room temperature for 1 h. Protein bands were detected using an enhanced chemiluminescence system (Thermo Fisher, USA). The grey scale of protein bands was analyzed using Image J software (National Institutes of Health, Bethesda, USA).

### Cell counting Kit-8 (CCK8) assay

For drug cytotoxicity assay, BHY and HSC3 cells transfected with si-NC or si-HMG20A were seeded into 96-well plates with 1, 2, 4, 8, 16 and 32 µM cisplatin treatment for 48 h. Then, 10 µl of the CCK8 solution (Solarbio, China) was added into each well and maintained at 37 °C for 2 h. Absorbance was measured at 450 nm by a microplate reader (Thermo Fisher, USA), and then the half-maximal inhibitory concentration (IC_50_) to cisplatin was calculated. For cell proliferation assay, 10 µl of the CCK8 solution was added into each well at 0, 24, 48 and 72 h. Absorbance at each time point were measured at 450 nm by a microplate reader (Thermo Fisher, USA).

### Transwell assay

Matrigel chambers (BD Biosciences, USA) were constructed according to manufacturer’s instructions. BHY and HSC3 cells transfected with si-NC or si-HMG20A were cultured in serum-free DMEM medium with 5 µM cisplatin, and then shifted to the upper Matrigel chambers (50 µL). Lower chambers were supplemented with DMEM containing 1% fetal bovine serum and 5 µM cisplatin (600 µL). After 48 h incubation at 37 °C, the cells on the upper surface of the membrane were removed, while the invaded cells on the lower surface were stained with 0.1% crystal violet for 20 min.

### Statistical analysis

All experiments were performed at least three replications. Data was presented as mean ± standard deviation. Statistical data were analyzed using GraphPad Prism 9.1.0. Student’s *t*-test was employed to compare two different groups. ANOVA followed by Turky’s test was employed to evaluate difference among multiple groups. The cutoff of statistical significance was P < 0.05.

## Results

### DNA repair capacity was positively correlated with the metastatic phenotype of OSCC

**Fig. 1:**
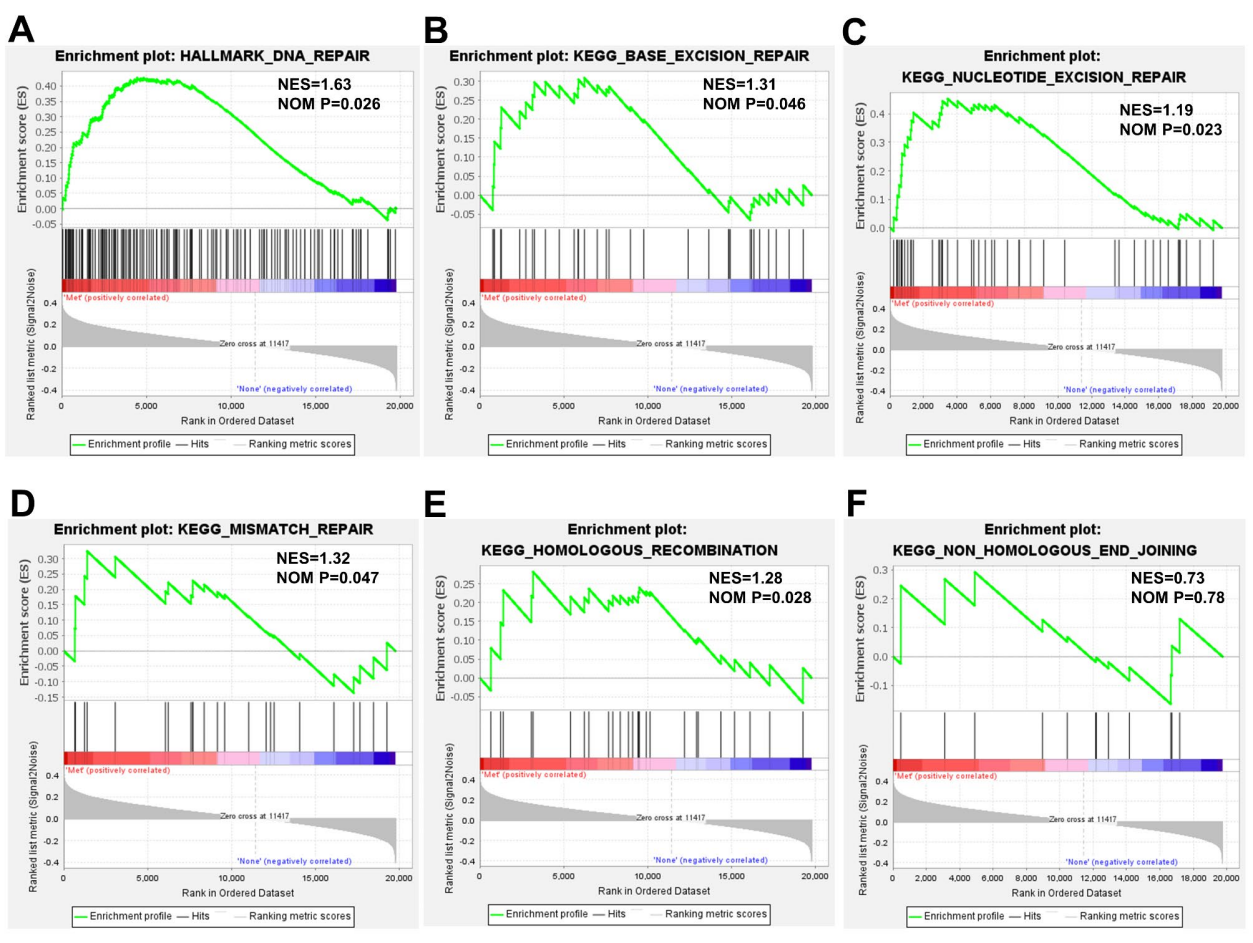
**DNA repair capacity was positively correlated with the metastatic phenotype of OSCC.** GSEA enrichment plots for “DNA repair” (A), “base excision repair” (B), “nucleotide excision repair” (C), “mismatch repair” (D), “homologous recombination” (E) and “non-homologous end-joining” (F). Met, metastatic OSCC group. None, non-metastatic OSCC group

Since metastasis is a key factor contributing to the poor prognosis of OSCC, GSEA was performed based on TCGA data in both metastatic and non-metastatic OSCC groups. GSEA enrichment plots of representative gene sets on DNA repair were shown in Fig. 1. “DNA repair” gene set was observed to have a significant positive correlation with the metastatic phenotype of OSCC (Fig. 1 A). DNA repair mainly includes base excision repair, nucleotide excision repair, mismatch repair, homologous recombination and non-homologous end-joining [10]. Hence, we investigated the differential activation of these five DNA repair pathways between metastatic and non-metastatic OSCC groups using GSEA. “Base excision repair”, “nucleotide excision repair”, “mismatch repair” and “homologous recombination” were significantly and positively correlated with the OSCC metastasis phenotype (Fig. 1B-E). There was also a positive correlation between “non-homologous end-joining” and metastasis phenotype, although the correlation was not significant (Fig. 1 F). As the enrichment in “base excision repair”, “nucleotide excision repair”, “mismatch repair” and “homologous recombination” was significant, while the enrichment in “non-homologous end-joining” was not significant, genes included in “base excision repair”, “nucleotide excision repair”, “mismatch repair” and “homologous recombination” gene sets were considered to be DNA repair-related genes. Taken together, DNA repair was positively correlated with the metastatic phenotype of OSCC.

### Clustering analysis of OSCC patients based on the expression of DNA repair-related genes

**Fig. 2:**
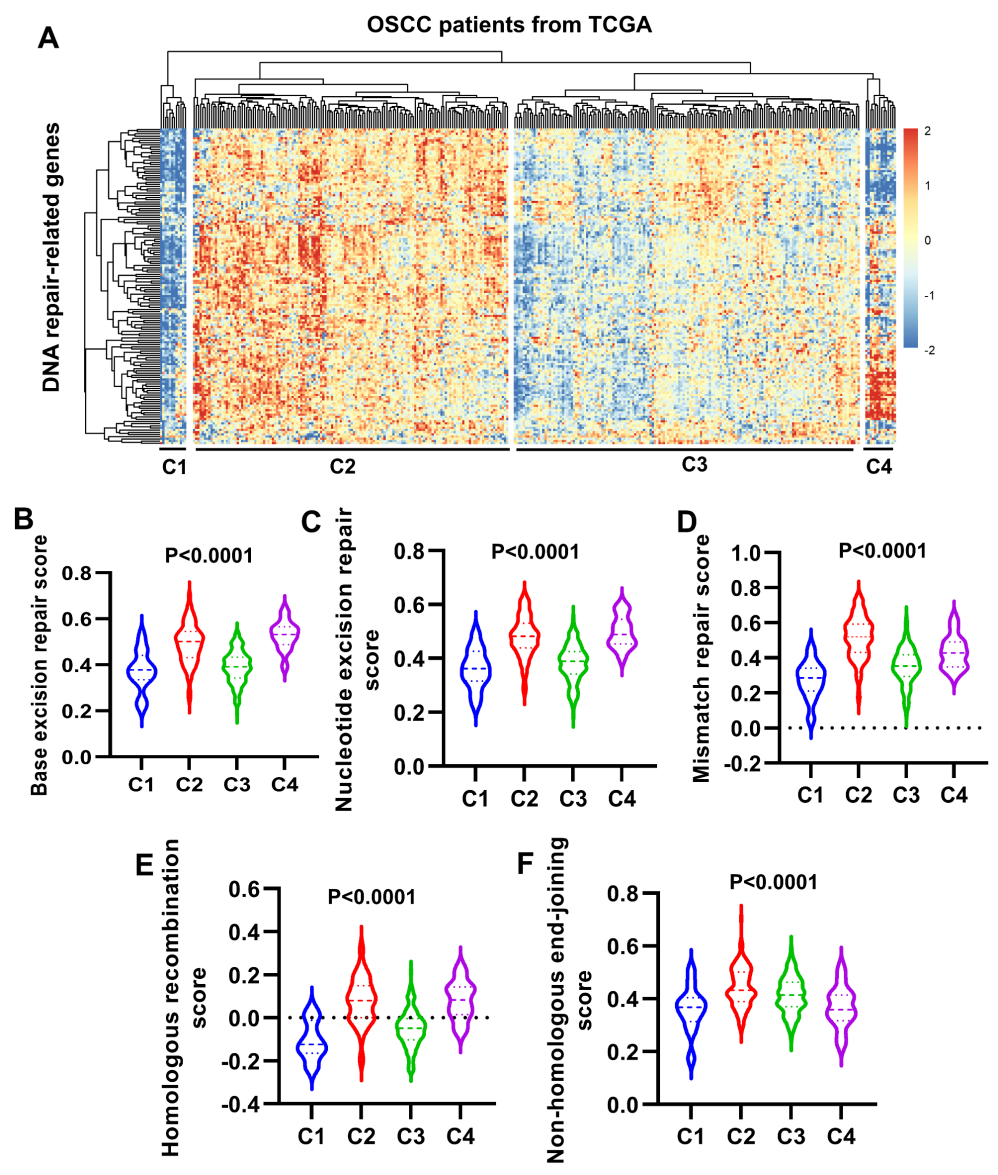
**Clustering analysis of OSCC patients based on the expression of DNA repair-related genes.** A, clustering of OSCC patients in TCGA database based on the expression of DNA repair-related genes. C1, cluster 1; C2, cluster 2; C3, cluster 3; C4, cluster 4. B-F, GSVA was applied to calculate base excision repair score (B), nucleotide excision repair score (C), mismatch repair score (D), homologous recombination score (E) and non-homologous end-joining score (F) of the four clusters. Comparison of differences among groups was performed using ANOVA followed by Turkey’s test

To further investigate the effects of DNA repair on OSCC progression, 328 OSCC patients from TCGA database were clustered according to the expression of DNA repair-related genes. As shown in Fig. 2 A, OSCC patients were clustered into four clusters, with C1 (n = 12) and C4 (n = 14) containing a smaller number of samples, and the majority of OSCC patients were clustered into C2 (n = 144) and C3 (n = 158). For the two clusters with large sample sizes, DNA repair-related genes were low expressed in C3, but high expressed in C2 (Fig. 2 A). Subsequently, base excision repair score, nucleotide excision repair score, mismatch repair score, homologous recombination score and non-homologous end-joining score for the four clusters were calculated using GSVA. Significant differences were found in all scores for C1, C2, C3 and C4 (Fig. 2B-F). It is noted that for the two clusters with a large sample size, all scores were higher for C2 than C3 (Fig. 2B-F). In total, OSCC patients were predominantly enriched in C2 and C3 based on the expression of DNA repair-related genes, and all DNA repair pathway scores of C2 were higher than those of C3.

### Clinical characteristics analysis of the four clusters

**Fig. 3:**
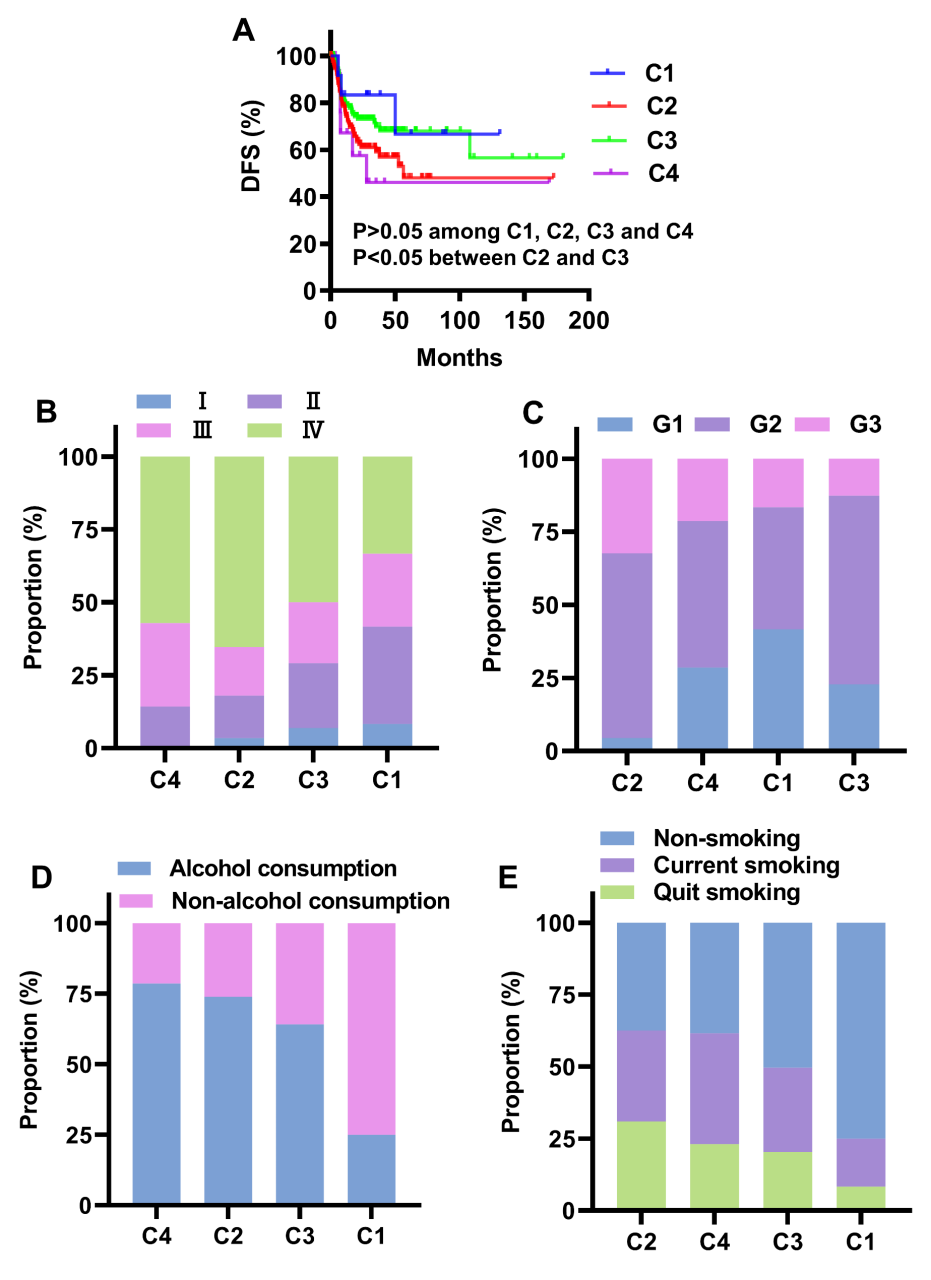
**Clinical characteristics analysis of the four clusters.** A, Disease-free survival (DFS) of C1, C2, C3 and C4. B-E, proportion of TNM stage (B), histological grading (C), alcohol consumption (D) and smoking (E) distribution of the four clusters. C1, cluster 1; C2, cluster 2; C3, cluster 3; C4, cluster 4. G1, highly differentiated OSCC; G2, moderately differentiated OSCC; G3, poorly differentiated OSCC.

Analysis of DFS in the four clusters revealed that DFS was poor in C2 and C4, and favorable in C1 and C3, while the difference in DFS among the four clusters was not significant (P > 0.05) (Fig. 3 A). Remarkably, DFS of C3 was significantly higher than C2 (P < 0.05) (Fig. 3 A).

Analysis of TNM stage revealed that the proportion of patients with advanced stages was 82% for C2 (16.7% for stage III, 65.3% for stage IV) and 70.9% for C3 (20.9% for stage III, 50.0% for stage IV), indicating that the proportion of patients with advanced stages (especially stage IV) was higher in C2 than C3 (Fig. 3B). The proportion of advanced patients in C1 was 58.3% (25% for stage III, 33.3% for stage IV), which was the lowest of the four clusters (Fig. 3B). The proportion of advanced patients in C4 was 85.7%, which was similar to that of C2 (Fig. 3B). Analysis of histological grading showed that the prevalence of poorly differentiated OSCC (G3) in descending order was 32.4% for C2, 21.4% for C4, 16.7% for C1 and 12.7% for C3 (Fig. 3 C).

Alcohol consumption and smoking are two key risk factors for OSCC. The distribution of alcohol consumption in C2 was higher than that in C3 (Fig. 3D). Although C1 and C4 had the lowest and highest percentages of alcohol consumption (25% for C1 and 78.6% for C4) respectively, the sample numbers contained in C1 and C4 were very small (Fig. 3D). As regards tobacco smoking, the proportion of smoking (including quit smoking and current smoking) in the four clusters ranged from 62.6% for C2, 61.6% for C4, 49.67% for C3 and 24.9% for C1 (Fig. 3E).

The above results indicated that for the two clusters with large sample sizes (C2 and C3), patients in C2 had poorer DFS, and higher proportion of advanced stages, poorly differentiated OSCC, alcohol consumption and smoking than C3.

### Identification of DNA repair-related genes controlled by metastatic-specific enhancers in OSCC

**Fig. 4:**
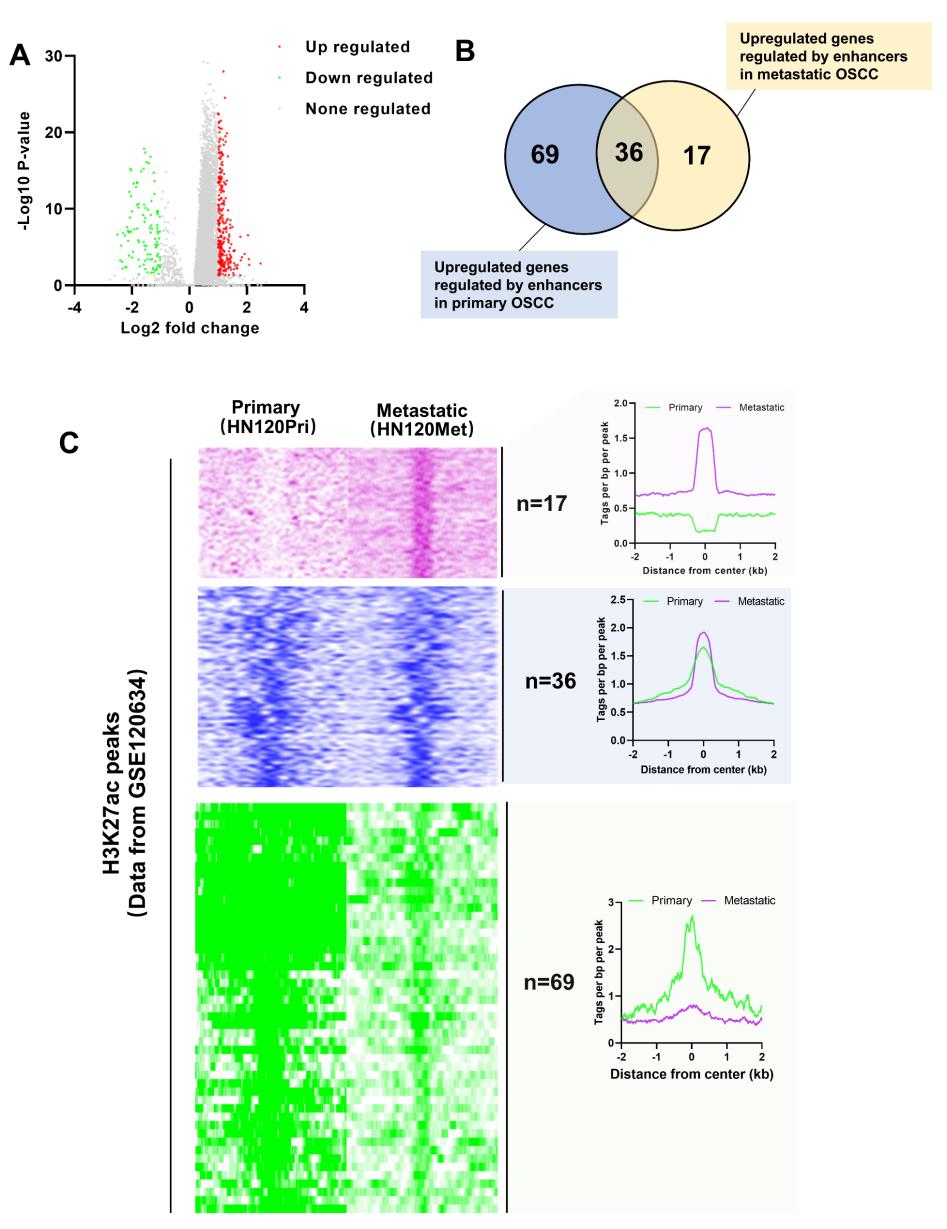
**Identification of DNA repair-related genes controlled by metastatic-specific enhancers in OSCC.** A, identification of differentially expressed DNA repair-related genes between cluster 2 and cluster 3 were performed based on TCGA. The cut-off values for differentially expressed genes were P < 0.05 and |log2 fold change|>1. B, overlapping analysis of upregulated genes controlled by enhancers in primary OSCC and metastatic OSCC. Enhancers of DNA repair-related genes in metastatic OSCC and primary OSCC were screened using H3K27ac ChIP-seq data of GSE120634. DNA repair-related gene located closest to an enhancer on the genome was defined as an enhancer-controlled DNA repair-related gene. C, H3K27ac peaks of metastatic-specific enhancers of DNA repair-related genes (n = 17), primary-specific enhancers of DNA repair-related genes (n = 69), and enhancers of DNA repair-related genes common to both metastatic OSCC and primary OSCC (n = 36) were shown in heatmap and aggregation plots

We focused on the differential expression of DNA repair-related genes between C2 and C3 due to the larger sample size of C2 and C3. Compared to C3, 283 genes were upregulated and 107 genes were downregulated in C2 (Fig. 4 A).

Subsequently, GSE120634 dataset was used to analyze enhancer-controlled genes in primary OSCC, and these enhancer-controlled genes were intersected with the upregulated genes in C2 to obtain 105 enhancer-regulated upregulated genes in primary OSCC (Fig. 4B). Similarly, enhancer-controlled genes in metastatic OSCC were identified using GSE120634. Taking the intersection of these genes with the upregulated genes in C2, 53 enhancer-controlled upregulated genes in metastatic OSCC were obtained (Fig. 4B). Overlapping analysis of enhancer-controlled upregulated genes in primary OSCC and metastatic OSCC found that 17 upregulated genes controlled by enhancers were specific to metastatic OSCC, 69 upregulated genes controlled by enhancers were specific to primary OSCC, and 36 enhancer-controlled genes were present in both metastatic OSCC and primary OSCC (Fig. 4B). H3K27ac signaling of metastatic-specific enhancers, primary-specific enhancers, and enhancers common to both metastatic OSCC and primary OSCC of DNA repair-related genes were shown in Fig. 4 C. The 17 DNA repair-related genes controlled by metastatic-specific enhancers were used for following exploration.

### HMG20A was screened as a key enhancer driver regulating DNA repair-related genes

**Fig. 5:**
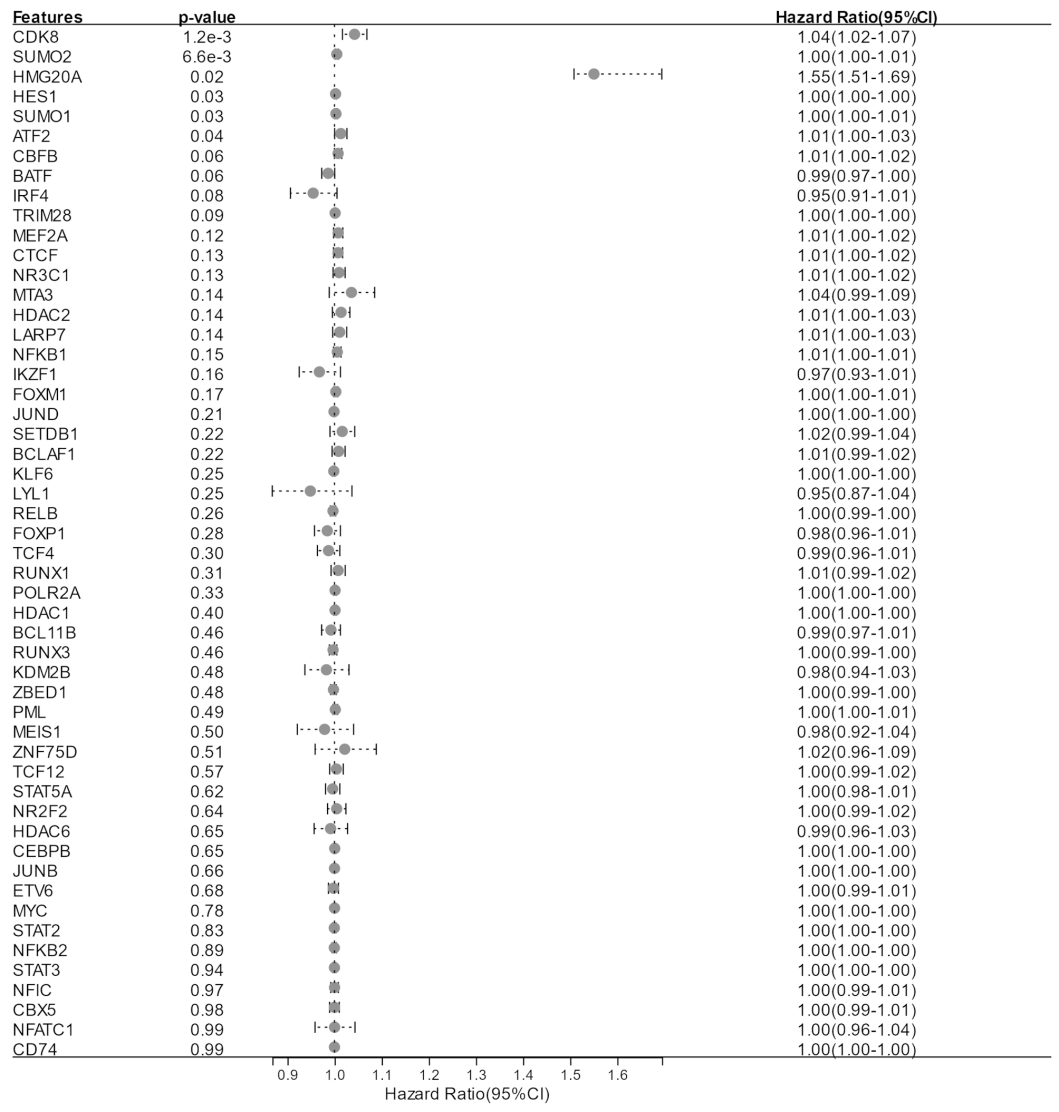
**Forest map of hazard ratio of the 52 transcription factors.** CI, confidence interval

**Fig. 6:**
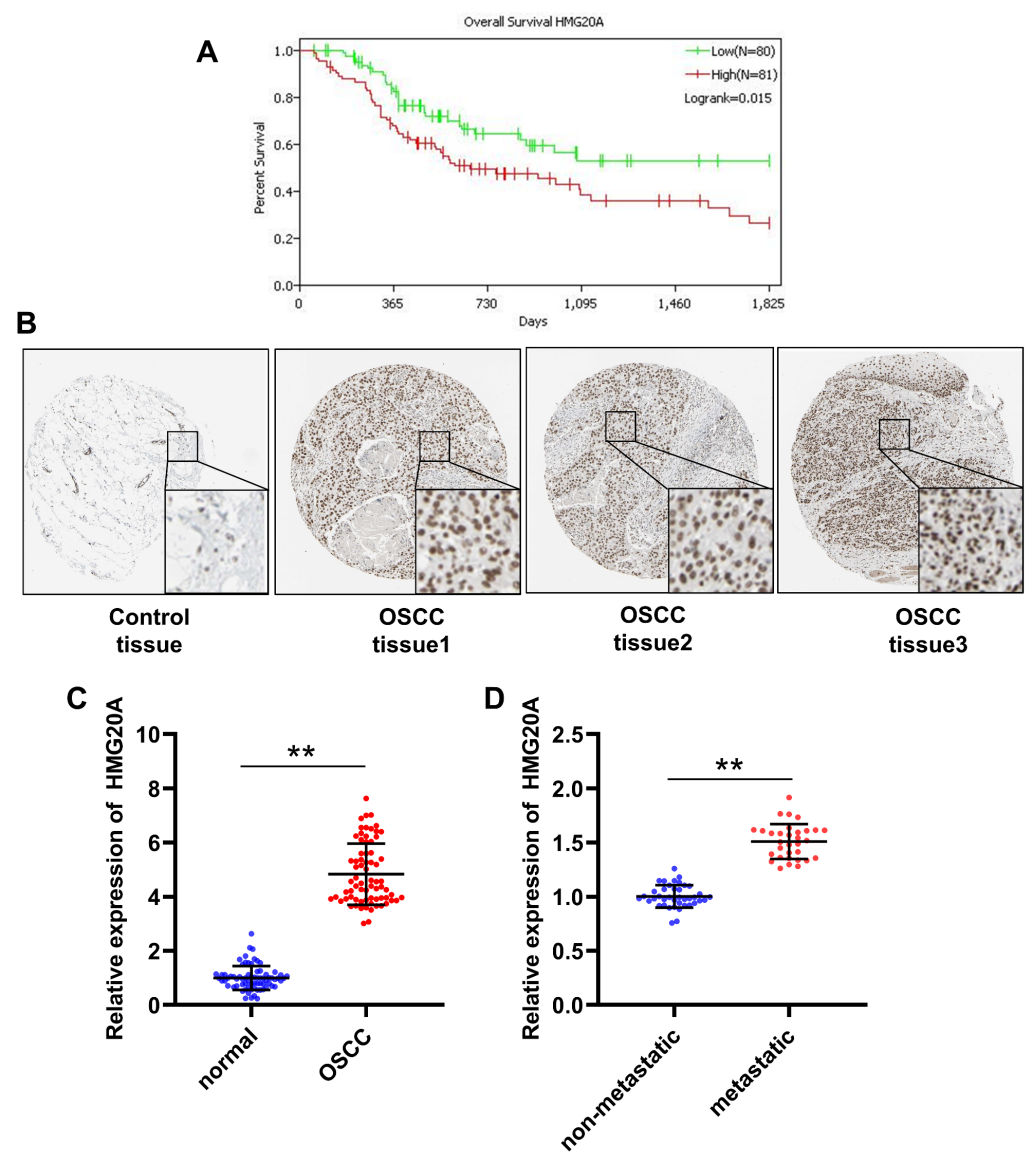
**HMG20A was screened as a key enhancer driver regulating DNA repair-related genes.** A, impact of HMG20A expression on overall survival in OSCC patients based on TCGA-OSCC cohort. Patients were classed in to HMG20A high expression and low expression groups according to the quartiles. B, HMG20A protein levels in OSCC and normal control tissues was analyzed using The Human Protein Atlas database. C, qRT-PCR was performed to measure HMG20A expression in 72 pairs OSCC and adjacent normal tissues collected in this study. **P < 0.01, normal group vs. OSCC group. D, qRT-PCR was performed to measure HMG20A expression in 40 non-metastatic and 32 metastatic OSCC tissues collected in this study. **P < 0.01, non-metastatic group vs. metastatic group

To explore the regulatory mechanisms of the 17 metastatic-specific enhancer-regulated DNA repair-related genes, transcription factors were predicted by the Toolkit for Cistrome Data Browser. A total of 60 potential transcription factors were predicted (Table S1). Relationship among overall survival and transcription factors expression in OSCC patients were analyzed based on TCGA-OSCC cohort. TPM < 1 was used as the threshold resulting 8 transcription factors with low expression in OSCC were removed. Subsequently, we calculated HRs and 95% CIs of the other 52 transcription factors to determine overall survival-related prognostic factors in OSCC patients. Only one transcription factor, HMG20A, was obtained using HR > 1.4 and P < 0.05 as screening criteria (Fig. 5). As shown in Fig. 6 A, the overall survival of OSCC patients with high HMG20A expression was significantly lower than that of patients with low HMG20A expression. According to the data from The Human Protein Atlas database, HMG20A expression was found to be obviously enhanced in OSCC tissues compared with control tissues (Fig. 6B). In addition, the expression of HMG20A in 72 pairs of OSCC (including 40 non-metastatic OSCC and 32 metastatic OSCC) and control tissues collected in this study was detected by qRT-PCR. Consistent with the results from The Human Protein Atlas database, the expression level of HMG20A in OSCC tissues was significantly higher than that in the control tissues (Fig. 6 C). Furthermore, HMG20A expression in metastatic OSCC tissues was significantly upregulated compared to non-metastatic OSCC tissues (Fig. 6D). Taken together, HMG20A was screened as a key enhancer driver for regulating DNA repair-related genes.

### HMG20A bound to metastatic-specific enhancers to regulate DNA repair-related genes expression

**Fig. 7:**
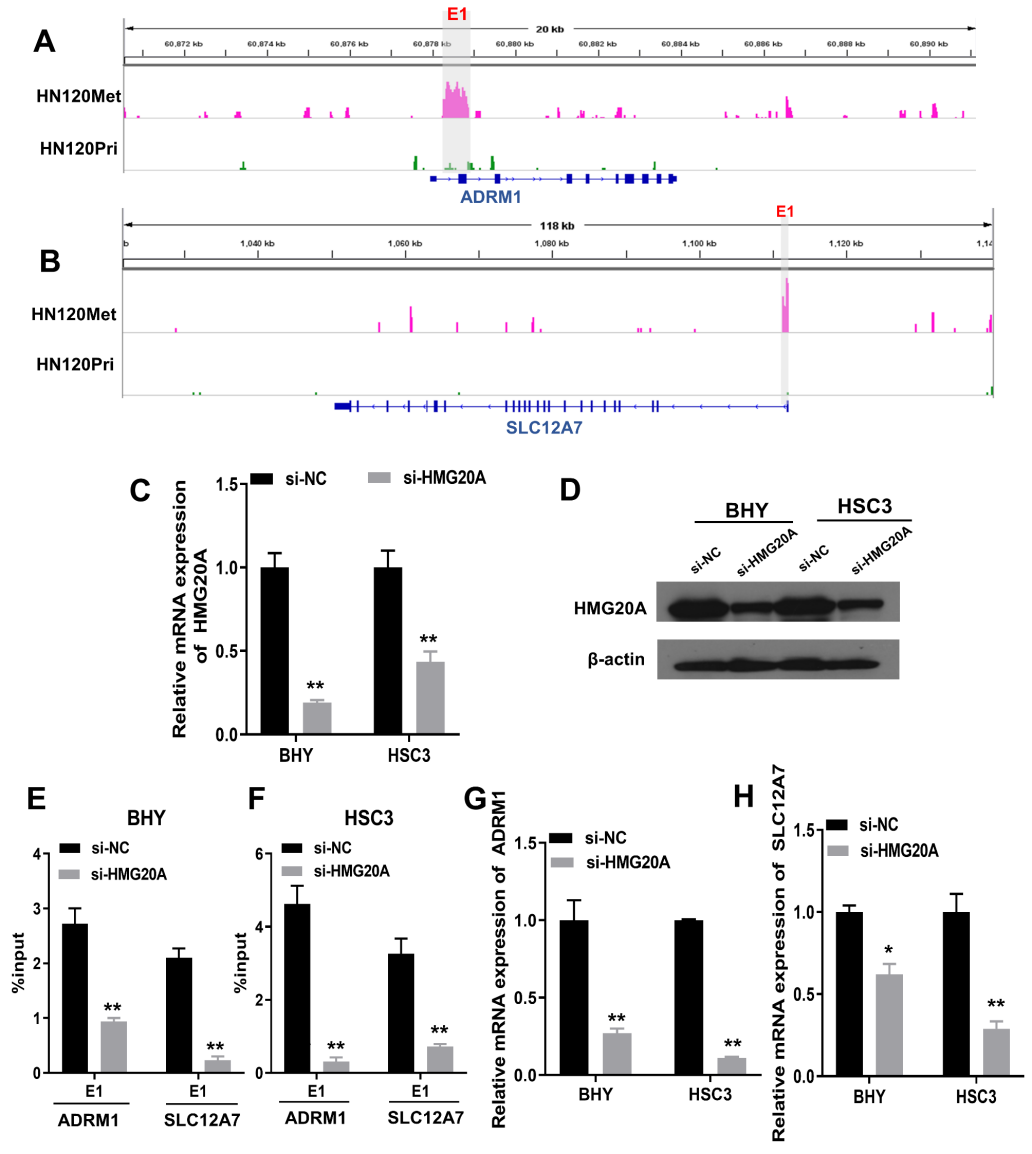
**HMG20A bound to metastatic-specific enhancers to regulate ADRM1 and SLC12A7 expression.** A/B, GSE120634 cohort was used for identification of enhancer regions (E1) around ADRM1 (A) and SLC12A7 (B) locus in HN120Met and HN120Pri cells. HN120Met is a metastatic OSCC cell line. HN120Pri is a primary OSCC cell line. C/D, BHY and HSC3 cells were transfected with si-HMG20A or si-NC. qRT-PCR and Western blotting were performed to measure mRNA (C) and protein (D) levels of HMG20A in si-HMG20A and si-NC groups. E/F, the status of H3K27ac modification in ADRM1 and SLC12A7 enhancer regions of BHY and HSC3 cells with or without HMG20A knockdown was examined by ChIP-qPCR. G/H, transcriptional levels of ADRM1 and SLC12A7 in BHY and HSC3 cells with or without HMG20A knockdown were detected by qRT-PCR. *P < 0.05, **P < 0.01, si-HMG20A group vs. si-NC group

To further validate the HMG20A regulation of metastatic-specific enhancer-regulated DNA repair genes, the top two upregulated genes in C2, ADRM1 and SLC12A7, were selected to validate the regulatory role of HMG20A. H3K27ac signals around ADRM1 and SLC12A7 locus on the genome of primary OSCC cells (HN120Pri) and metastatic OSCC cells (HN120Met) were analyzed using GSE120634. A region of significantly elevated H3K27ac signal was present around ADRM1 locus in HN120Met cells (E1), but no elevated H3K27ac region was present around ADRM1 locus in HN120Pri cells (Fig. 7 A). Similarly, a region of enhanced H3K27ac signaling was present around SLC12A7 locus in HN120Met cells (E1), whereas absent in the same region in HN120Pri cells (Fig. 7B). These results suggested that the metastatic-specific enhancers exist around ADRM1 and SLC12A7 locus in metastatic OSCC cells. Subsequently, we investigated whether these candidate enhancers were able to bind to HMG20A. We knocked down the expression of HMG20A in BHY and HSC3 cells, and demonstrated that transfection with si-HMG20A resulted in a significant knockdown of HMG20A expression at both the transcriptional and translation levels (Fig. 7 C, 7D). Transfection with si-HMG20A significantly inhibited the H3K27ac modification in the enhancer regions of ADRM1 and SLC12A7 (Fig. 7E F). Furthermore, we noted a significant decrease in transcriptional levels of ADRM1 and SLC12A7 after HMG20A knockdown (Fig. 7G H). Altogether, these data indicated HMG20G bind to metastatic-specific enhancers and thus contribute to the expression of DNA repair-related genes.

### Knockdown of HMG20A enhanced cisplatin sensitivity, and inhibited DNA damage repair, proliferation and invasion of OSCC cells

**Fig. 8:**
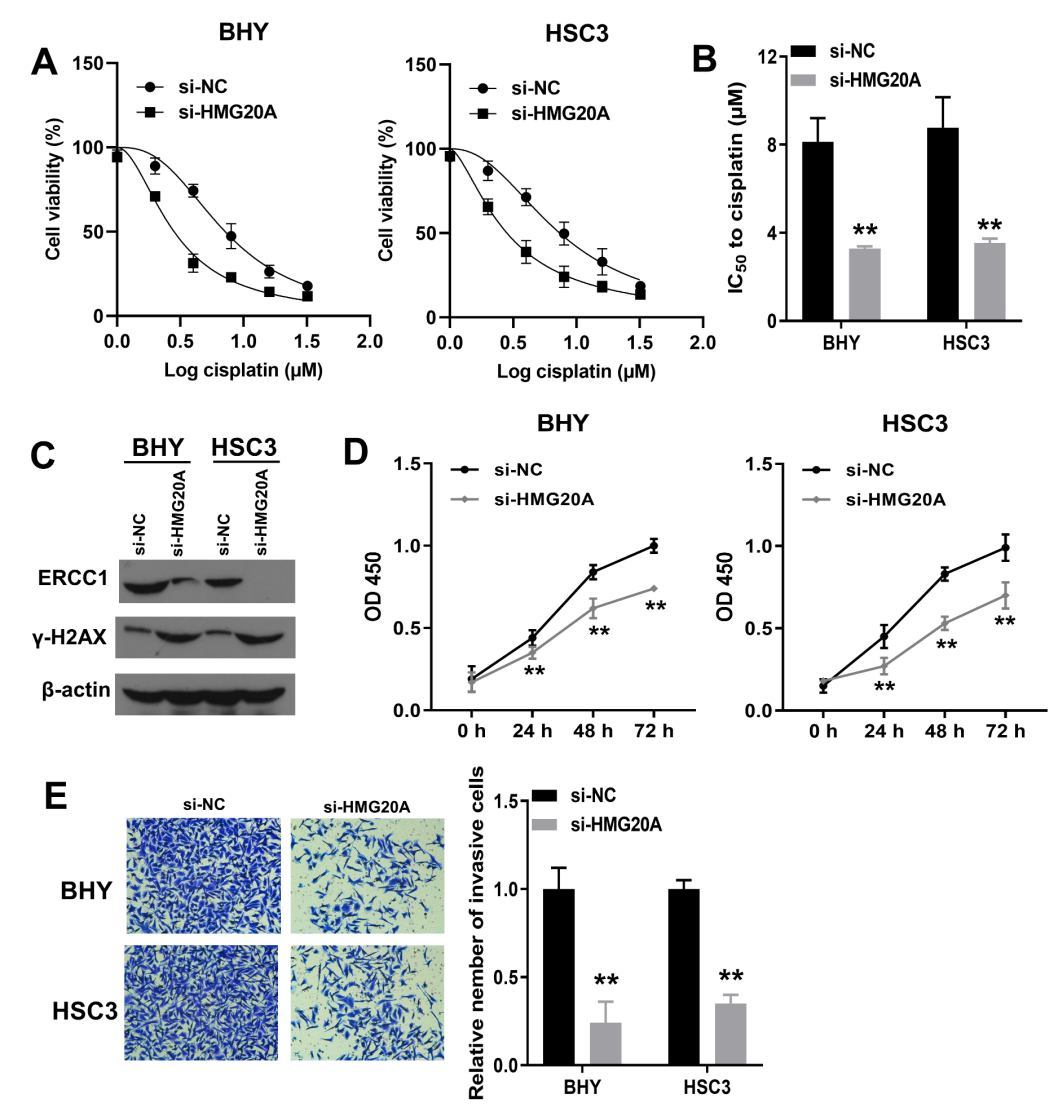
**Knockdown of HMG20A enhanced cisplatin sensitivity, and inhibited DNA damage repair, proliferation and invasion of OSCC cells.** A, cell viability of BHY and HSC3 cells transfected with si-HMG20A or si-NC at different cisplatin concentrations. B, IC_50_ to cisplatin of BHY and HSC3 cells transfected with si-HMG20A or si-NC. C, BHY and HSC3 cells transfected with si-HMG20A or si-NC were treated with 5 µM cisplatin for 48 h. Western blotting was used to measure the protein levels of ERCC1 and γ-H2AX. β-actin was the internal control. D/E, BHY and HSC3 cells transfected with si-HMG20A or si-NC were treated with 5 µM cisplatin for 48 h. CCK8 and Transwell assay was used to detect cell proliferation (D) and invasion (E). **P < 0.01, si-HMG20A group vs. si-NC group

We determined the effect of HMG20A knockdown on cisplatin sensitivity of OSCC cells. Compared with cells transfected with si-NC, medium with different doses of cisplatin inhibited the viability of cells transfected with si-HMG20A (Fig. 8 A). Transfection of si-HMG20A resulted in a distinct decrease in IC_50_ to cisplatin in both BHY and HSC3 cells (Fig. 8B). The IC_50_ to cisplatin of BHY cells transfected with si-NC, BHY cells transfected with si-HMG20A, HSC3 cells transfected with si-NC and HSC3 cells transfected with si-HMG20A was 8.13, 3.27, 8.77 and 3.53 µM, respectively (Fig. 8B). Therefore, we selected a cisplatin concentration of 5 µM for subsequent studies.

To verify the roles of HMG20A on DNA damage repair, BHY and HSC3 cells with or without HMG20A knockdown were treated with 5 µM cisplatin for 48 h, and then the effects of HMG20A knockdown on cell phenotype was examined. The expression level of DNA damage response protein, γ-H2AX, was significantly increased in HMG20A knockdown cells compared with the control cells (Fig. 8 C). However, the expression level of DNA repair protein, ERCC1, was significantly decreased after HMG20A knockdown (Fig. 8 C). Compared with the si-NC group, transfection with si-HMG20A significantly inhibited the proliferation of BHY and HSC3 cells treated with cisplatin (Fig. 8D). Additionally, under cisplatin condition, the number of invasive cells significantly decreased after knockdown of HMG20A in both BHY and HSC3 cells (Fig. 8E). Taken together, HMG20A knockdown enhanced cisplatin sensitivity of OSCC cells. Knockdown of HMG20A inhibited OSCC cells from repairing DNA damage caused by cisplatin, as well as proliferation and invasion of OSCC cells.

## Discussion

Factors contributing to DNA damage can be divided into two categories, endogenous factors and exogenous factors [25]. Endogenous factors refer to DNA damage caused by by-products of cellular metabolism (e.g. reactive oxygen species) and factors such as base mismatches, insertions or deletion during DNA replication [25]. Exogenous factors mainly include the ultraviolet light, ionizing radiation, chemotherapeutic drugs, etc. [25]. DNA repair is essential to maintain cellular homeostasis. Many anti-cancer drugs achieve the purpose of treatment by inducing DNA damage of tumor cells. However, tumor cells can activate DNA repair mechanism to repair the damage, resulting in drug resistant [11, 26]. Blocking DNA repair pathways is crucial to improve the therapeutic efficiency of OSCC. Elucidating the molecular regulatory mechanisms of DNA repair has implications for improving the efficacy of tumor chemotherapy. In this study, we analyzed the relationship between the expression of DNA repair-related genes and metastasis, prognosis, staging, differentiation, and risk factors (alcohol consumption and smoking) of OSCC. Metastatic-specific enhancer-regulated DNA repair-related genes were screened, and transcription factors of these genes were predicted. Furthermore, the effects of the key transcription factor on metastatic-specific enhancers-controlled target genes were verified, and the impacts of the key enhancer driver on DNA repair, proliferation and invasion of OSCC cells under cisplatin treatment were explored in vitro.

The main DNA repair pathways triggered by DNA damage include base excision repair, nucleotide excision repair, mismatch repair, homologous recombination and non-homologous end-joining [10]. It is well known that metastasis is one of the major reasons for the poor prognosis of OSCC [3, 7]. Therefore, we analyzed the enrichment of metastatic OSCC and non-metastatic OSCC in the above five DNA repair pathways using GSEA, and found that all DNA repair pathways were positively correlated with the metastatic phenotype of OSCC, although the correlation between non-homologous end-joining and metastatic phenotype was not significant. OSCC patients were clustered according to the expression of DNA repair-related genes resulted in 4 clusters (C1, C2, C3 and C4). Since the sample size of C1 and C4 was small, we focused on the two clusters with a large sample size, C2 (cluster with high expression of DNA repair-related genes) and C3 (cluster with low expression of DNA repair-related genes). Base excision repair score, nucleotide excision repair score, mismatch repair score, homologous recombination score and non-homologous end-joining score of C2 were higher than those of C3. DFS of patients in C2 was significantly worse than that in C3. Tumor stage and differentiation are important prognostic factors [27, 28]. We analyzed the distribution of tumor stage and differentiation of the four clusters, and found that the proportions of patients with advanced stage and low-differentiation were higher in C2 than in C3. In addition, we also considered two important risk factors, alcohol consumption and smoking [1, 3]. The results showed that the proportion of patients with alcohol consumption and smoking was higher in C2 than in C3. These results suggested that alcohol consumption and smoking may affect DNA repair of OSCC cells, and abnormal DNA repair of OSCC cells was closely related to metastasis, tumor stage, differentiation, and adversely affects the prognosis of OSCC patients.

To further investigate the regulatory mechanisms of DNA repair, we identified differentially expressed DNA repair-related genes between C2 and C3. A total of 390 differentially expressed genes were screened, of which 283 were upregulated in C2. Aberrant regulation of gene expression by enhancers is a key regulatory mechanism in cancer progression. Enhancers are DNA sequences that regulate gene expression, and contain sequence-specific transcription factor recognition and binding sites, which bind to transcription factors and initiate transcription of target genes [18]. In recent years, the mechanism of action of enhancers has been continuously explored. Active enhancers are located in open chromatin regions, with the high degree of H3K27ac modification being one of their distinguishing features [29, 30]. In the present study, we identified enhancer-controlled upregulated DNA repair-related genes in primary OSCC cells and metastatic OSCC cells. A total of 17 metastatic-specific enhancer-controlled upregulated DNA repair-related genes were screened out. Function of enhancers in promoting the expression of target genes is dependent on the binding of transcription factors [18]. To explore the regulatory mechanisms of the 17 metastatic-specific enhancer-controlled upregulated genes, we predicted their transcription factors, and analyzed the impacts of transcription factors on the overall survival of OSCC patients. We found that only the expression of HMG20A had a significant impact on the overall survival of OSCC patients, exhibiting that high expression of HMG20A corresponded with a poor overall survival. The expression of HMG20A was significantly higher in OSCC tissues than in normal control tissues. In addition, HMG20A expression was upregulated in metastatic OSCC tissues compared to non-metastatic OSCC tissues.

High mobility group 20 A (HMG20A), also known as HMGX1 or HMGXB1, maps to chromosome 15q24 and is homologous to HMG20B [31, 32]. HMG20B is the core subunit of the Lys-specific demethylase 1/REST co-repressor 1 (LSD1-CoREST) histone demethylase complex, and HMG20A can function in place of HMG20B [32]. It has been reported that HMG20A has important biological functions such as promoting functional maturation of pancreatic β-cells, promoting neuronal differentiation, regulating inflammatory responses and epithelial mesenchymal transition [32–34]. A prognostic model of SUMOylation-regulated genes involving HMG20A could predict the prognosis of OSCC [35]. However, the role and mechanism by which HMG20A regulates OSCC remains largely unknown. In this study, we selected two genes, ADRM1 and SLC12A7, which were the top two upregulated genes in C2, for validation. ADRM1 is involved in proteasome composition and acts as a ubiquitin receptor to recruit deubiquitinating enzymes [36, 37]. Aberrant expression of ADRM1 is associated with a variety of cancers [38–41]. SLC12A7, also known as KCC4, is involved in cell volume homeostasis, inorganic ion homeostasis and transmembrane transport [42–44]. In the present study, we found the presence of metastatic-specific enhancer regions of ADRM1 and SLC12A7 locus in metastatic OSCC cells, which were absent in primary OSCC cells. Furthermore, we verified the binding of HMG20A to metastatic-specific enhancers of ADRM1 and SLC12A7 by ChIP-qPCR. Knockdown of HMG20A inhibited the expression of ADRM1 and SLC12A7 in metastatic OSCC cells.

Cisplatin-based chemotherapy is the first-line chemotherapy agent for OSCC [12]. Cisplatin covalently binds to the N7 position of the purine base of DNA, forming adducts such as intra-strand cross links, which trigger cytotoxicity [45]. In the present study, we found that cisplatin sensitivity of metastatic OSCC cells was enhanced after knockdown of HMG20A. Excision of cisplatin-DNA adducts by the nucleotide excision repair pathway is the primary method of cellular repair of DNA damage caused by cisplatin [13]. ERCC1 is an essential factor in nucleotide excision repair pathway [13]. Expression of ERCC1 is associated with chemoresistance in many cancers [46, 47]. In the present study, we demonstrated that HMG20A knockdown inhibited ERCC1 protein expression in BHY and HSC3 cells under cisplatin treatment. In addition, DNA damage caused by cisplatin can be repaired by homologous recombination and non-homologous end-joining, among which homologous recombination repair has a more stringent repair mechanism, thus ensuring a high degree of accuracy [48–50]. When a DNA double strand break occurs, γ-H2AX is enriched at the break sites [16]. Subsequently, RAD51 was recruited to the γ-H2AX-labeled fracture site mediated by BRCA1 [51]. RAD51 searches for homologous DNA sequences along the sister chromatids, mediates the linking of sister chromatids by single-stranded nucleotides at the 3’ end, and then DNA polymerase resynthesizes the excised nucleotide sequence using the sister chromatids as a template [51]. In the present study, we found that knockdown of HMG20A resulted in upregulation of γ-H2AX. These results suggested that HMG20A expression facilitates OSCC cells to resist DNA damage caused by cisplatin. Furthermore, we demonstrated that knockdown of HMG20A inhibited the proliferation and invasion of OSCC cells under cisplatin treatment. The detailed mechanism by which HMG20A regulates DNA repair in OSCC cells will be explored in further study.

## Conclusion

The overall data of this work found that HMG20A was a key enhancer driver that regulated DNA repair-related genes in OSCC cells, and bound to metastatic-specific enhancers to promote target gene expression in metastatic OSCC cells. Knockdown of HMG20A in metastatic OSCC cells enhanced cisplatin sensitivity, inhibited the repair of cisplatin-induced DNA damage, and suppressed proliferation and invasion of OSCC cells. HMG20A has the potential to be a target for blocking DNA repair in OSCC cells, and is expected to be a promising novel target for clinical treatment of OSCC.

## Electronic supplementary material

Below is the link to the electronic supplementary material.

Supplementary Table S1 Potential transcription factors of 17 metastatic-specific enhancer-regulated DNA repair-related genes.

Supplementary Figure S1 Original images of Western blotting.

## Data Availability

Data for this study can be obtained under reasonable conditions by contacting QIU Yongle.
