# Supplementary material for: HMG20A was identified as a key enhancer driver associated with DNA damage repair in oral squamous cell carcinomas
Source: BMC Oral Health. 2022 Nov 5;22:473. doi: 10.1186/s12903-022-02500-y (PMC9636648; doi:10.1186/s12903-022-02500-y)
Supplement: Supplementary file 1 — Supplementary Table S1 Potential transcription factors of 17 metastatic-specific enhancer-regulated DNA repair-related genes. [file 12903_2022_2500_MOESM1_ESM.docx]

**Table S1** Potential transcription factors of 17 metastatic-specific enhancer-regulated DNA repair-related genes.

| Factor | GIGGLE_score |
| --- | --- |
| ATF2 | 17.24696 |
| BATF | 18.26023 |
| BCL11B | 17.88978 |
| BCLAF1 | 17.4632 |
| CBFB | 18.28789 |
| CBX5 | 18.33851 |
| CD74 | 18.408 |
| CDK8 | 17.09885 |
| CEBPB | 18.60698 |
| CTCF | 18.68165 |
| ESR1 | 19.34614 |
| ETV6 | 17.59498 |
| FOXA2 | 17.7654 |
| FOXM1 | 17.49747 |
| FOXP1 | 19.62506 |
| HDAC1 | 16.79419 |
| HDAC2 | 17.86904 |
| HDAC6 | 18.78952 |
| HES1 | 19.88714 |
| HMG20A | 17.98537 |
| HOXA9 | 18.88856 |
| IKZF1 | 16.19443 |
| IRF4 | 18.204 |
| JUNB | 17.67183 |
| JUND | 18.95132 |
| KDM2B | 16.96245 |
| KLF6 | 15.77879 |
| LARP7 | 18.59932 |
| LYL1 | 19.14435 |
| MEF2A | 18.37554 |
| MEIS1 | 19.54778 |
| MTA3 | 17.01654 |
| MYC | 18.93268 |
| NFATC1 | 42.33676 |
| NFIC | 16.97624 |
| NFKB1 | 18.67393 |
| NFKB2 | 19.07436 |
| NKX2-1 | 17.65607 |
| NR2F2 | 18.3791 |
| NR3C1 | 18.51748 |
| PML | 16.53818 |
| POLR2A | 18.59325 |
| PPARG | 18.04268 |
| PR | 17.81722 |
| RELB | 19.84465 |
| RUNX1 | 17.31722 |
| RUNX3 | 17.78825 |
| SCRT1 | 17.49841 |
| SETDB1 | 19.37195 |
| STAT2 | 18.71597 |
| STAT3 | 18.35704 |
| STAT5A | 17.46208 |
| SUMO1 | 18.99503 |
| SUMO2 | 18.82523 |
| TCF12 | 19.14927 |
| TCF4 | 18.31681 |
| TRIM28 | 18.94771 |
| ZBED1 | 17.83673 |
| ZNF736 | 17.24174 |
| ZNF75D | 18.53272 |
