# Supplementary figures and images for: HMG20A was identified as a key enhancer driver associated with DNA damage repair in oral squamous cell carcinomas
Source: BMC Oral Health. 2022 Nov 5;22:473. doi: 10.1186/s12903-022-02500-y (PMC9636648; doi:10.1186/s12903-022-02500-y)

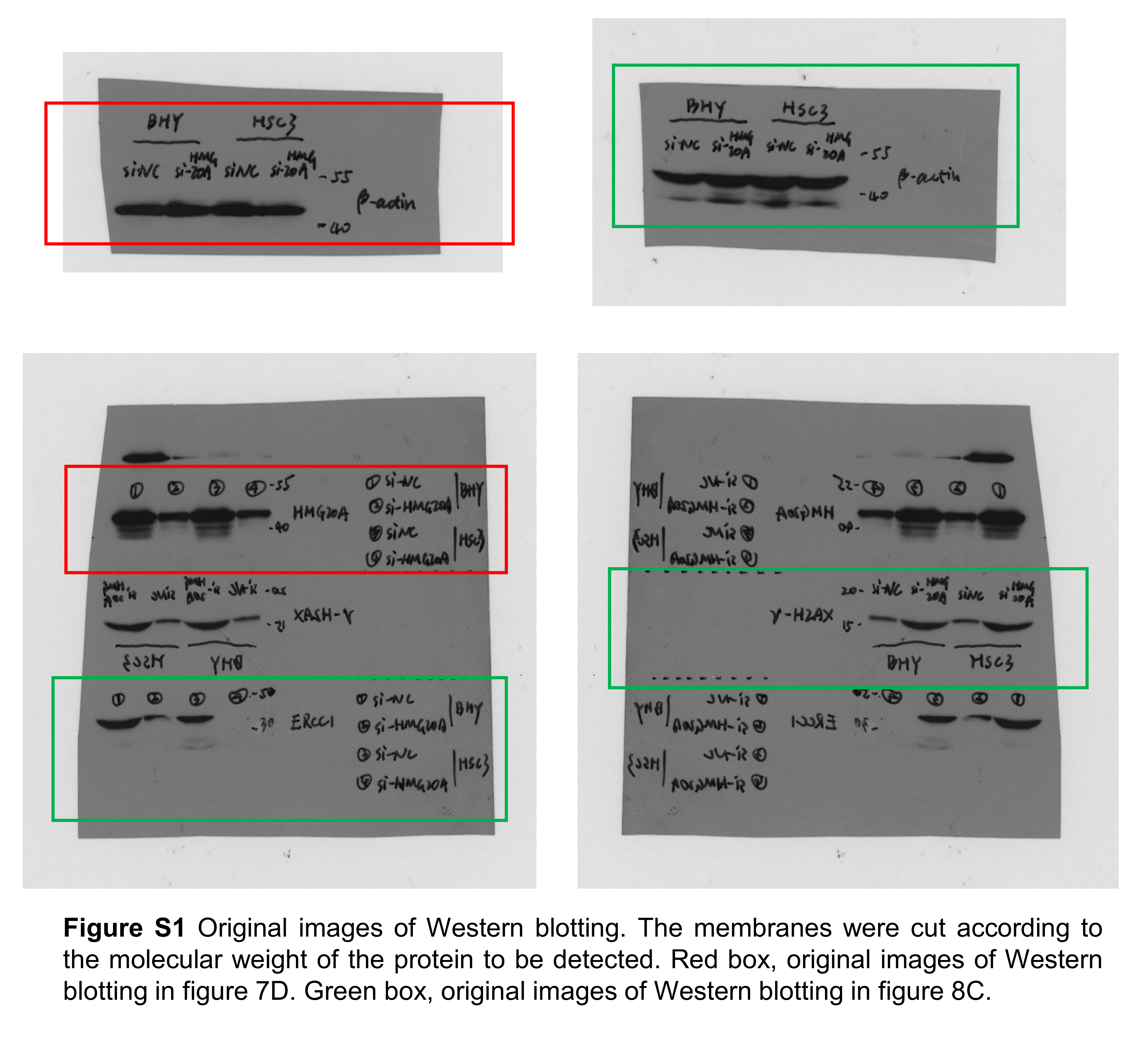

Supplement: Supplementary file 2 — Supplementary Figure S1 Original images of Western blotting. [file 12903_2022_2500_MOESM2_ESM.tif]
